# Human‐Like Sensing and Reflexes of Graphene‐Based Films

**DOI:** 10.1002/advs.201600130

**Published:** 2016-06-13

**Authors:** Qin Zhang, Lifang Tan, Yunxu Chen, Tao Zhang, Wenjie Wang, Zhongfan Liu, Lei Fu

**Affiliations:** ^1^ College of Chemistry and Molecular ScienceWuhan UniversityWuhan430072P. R. China; ^2^ Center for NanochemistryCollege of Chemistry and Molecular EngineeringPeking UniversityBeijing100871P. R. China

**Keywords:** human‐like sensing, reflex, graphene, stimulation, mechanical motion

## Abstract

Humans have numerous senses, wherein vision, hearing, smell, taste, and touch are considered as the five conventionally acknowledged senses. Triggered by light, sound, or other physical stimulations, the sensory organs of human body are excited, leading to the transformation of the afferent energy into neural activity. Also converting other signals into electronical signals, graphene‐based film shows its inherent advantages in responding to the tiny stimulations. In this review, the human‐like senses and reflexes of graphene‐based films are presented. The review starts with the brief discussions about the preparation and optimization of graphene‐based film, as where as its new progress in synthesis method, transfer operation, film‐formation technologies and optimization techniques. Various human‐like senses of graphene‐based film and their recent advancements are then summarized, including light‐sensitive devices, acoustic devices, gas sensors, biomolecules and wearable devices. Similar to the reflex action of humans, graphene‐based film also exhibits reflex when under thermal radiation and light actuation. Finally, the current challenges associated with human‐like applications are discussed to help guide the future research on graphene films. At last, the future opportunities lie in the new applicable human‐like senses and the integration of multiple senses that can raise a revolution in bionic devices.

## 1 Introduction

Have you ever seen a baby catch something and then bite it without hesitation? Don't worry. It just wants to understand this world with its sensory organs directly. Sensory receptors, neural circuits, as well as parts of the brain involved in sensory perception are included in a sensing system. It is generally thought that sensing systems are those known as vision, hearing, olfaction (smell), taste, and somatic sensation (touch). Through a process of light, sound or other physical stimulations, the object excites the sensory organs of the human body, leading to the transformation of the afferent energy into neural activity.

What should be first mentioned is the visual perception, the capacity of elucidating the surroundings via dealing with the information contained in visible light. Retina, the light‐sensitive organ in the back of the eye, is actually an isolated part of the brain serving as a transducer which converts light patterns into neuronal signals. Also transforming light signals to electronical signals, the solar cell (SC) and the photodetector (PD) are photovoltaic regardless of whether the sunlight or an artificial light serves as the source. The second sensory organ is the hearing, which perceives the sound by sensing vibrations, pressure variation of the surrounding medium with time. The eardrum would detect the vibrations, also known as mechanical waves, and then transduce them into nerve impulses. Based on this mechanism, people also developed a series of high‐performance devices which will potentially replace the current techniques, such microphone, loudspeaker, and earphone. The third sense is olfaction, the sense of smell, which is reconciled by specialized sensory cells in the nasal cavity. When odorant molecules bind to specific sites on the olfactory receptors, olfaction occurs and thus detects the presence of smell. Similar to the binding between odor and olfaction receptor neuron, gas sensor would adsorb gas molecules on the surface and induce conductance changes. The forth sensory organ is gustation, also within the five traditional senses, sensing the food or other substances on the tongue. It is the sensation produced when a substance in the mouth reacts chemically with taste receptor cells. Corresponding to the chemical reaction between a substance and taste receptor cells, the biosensor adsorbs the biomacromolecules and react with them. The last sensory organ is the tactile sense or mechanoreceptor, actively exploring surfaces and objects by a subject in movement rather than passive contact of a subject in a static state. This theory would be similar to the emerging devices, such as touch screen, electronical skin, and wearable devices. Additionally, the reflex is a reflection of the cerebral cortex which involves the most diverse stimuli utilized to different sensory areas. A reflex action, different from a reflex, is considered as an unwitting and nearly instantaneous behavior reacting to a stimulus. In other words, the organism will move or act when stimulated. Working the same way, actuated devices would also mechanically respond to the stimulations, such as thermal radiation and photostimulation.

Similar to the human sensory organs mentioned above, graphene‐based film shows its great potential in future applications, for instance, light‐sensitive devices, gas sensors and touch devices. As a prevailing member of carbon nanomaterials, graphene has enormous influence on various fields, such as physics and chemistry, materials science and engineering, increasingly transforming into a host of interdisciplinary advances in nanotechnology.^1^, ^2^ The high mobility,^3^ transparency^4^ and flexibility^5^ of graphene all contribute to its appeal. Thereinto, the transmittance of a single layer graphene is high in the extreme (97.7%).^4^ In addition, the outstanding mechanical strength and chemical stability, combined with cost‐effective production, make graphene a top choice for flexible sensory devices.

**Scheme 1:**
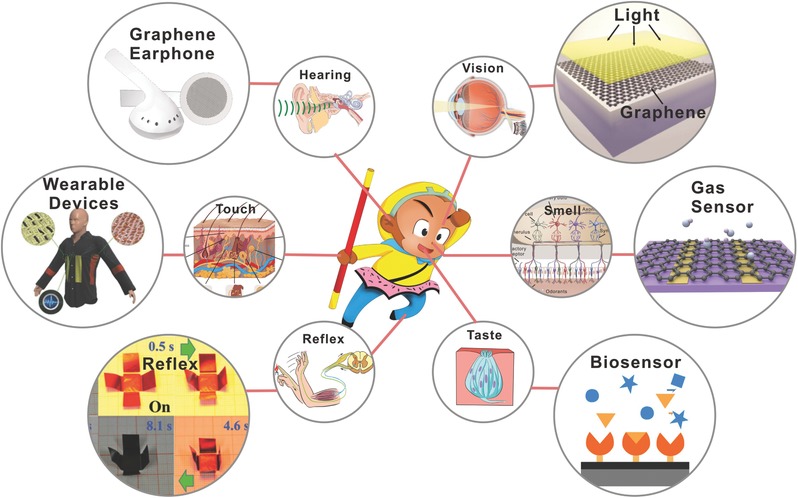
Schematic diagram of human‐like senses and reflexes of graphene‐based film.

During the last few years, numerous advances have been managed in the flexible graphene films with higher quality, greater conductivity and higher transparency. In this review, we provide a summary of current developments focusing on the synthesis, preparation and optimization of graphene film. As is illustrated in **Scheme** **1**, we focused on reviewing the human‐like senses and reflexes of graphene‐based films. Corresponding to the five senses (vision, hearing, olfaction, gustation, tactile) we human beings have, graphene‐based films show inherent advantages in responding to tiny stimulations; for instance, light, sound, smell, taste, and touch. And the responses of graphene‐based film make it suitable for a preponderance of light‐sensitive devices, acoustic devices, gas sensors, biosensors and wearable devices. Along with the reflex action of human, similarly, graphene‐based film also exhibits reflex actions when being simulated, such as thermal radiation and light actuated. Due to the complexity of the human body structure, graphene‐based films still have largely unexplored space to offer. Before closing, besides the challenges and future prospects of graphene‐based films in constant human‐like senses, more interesting and untapped potential of graphene‐based films is definitely worth looking forward to.

## 2 Properties and Preparation

### 2.1 Properties

Similar to the several human organs, the graphene‐based devices mentioned above were also anticipated having excellent performance. Thus the ideal graphene is desperately needed. Compared to the traditional materials, such as conductive glass, the monolayer graphene has extremely low sheet resistance of 30 Ω sq^–1^ in principle, whose transparency was up to 97.7%. Moreover, graphene is also a good thermal conductive material. The thermal conductivity of monolayer graphene is up to 5.30 ± 0.48 × 10^3^ W mK^–1^. And the excellent mechanical properties and flexibility of graphene also give it a more promising future of applications, such as serving as flexible sensors and detectors.

In our human body, neurotransmitters are regarded as the messenger in synaptic transmission, which is essential for the human internal signal transmission. This is comparable to the transfer of the carriers in graphene, which are also delivering the electronic information of graphene. The electrons and holes of graphene in concentrations are up to 10^13^ cm^–2^ and the mobility is up to 10 000 cm^2^ V^–1^ s^–1^ in room temperature and 200 000 cm^2^ V^–1^ s^–1^ at low temperature.^3^, ^6^ For applications of graphene, the sheet resistance is a key evaluation index of conductivity. As the above mentioned, the theoretical value of the sheet resistance of monolayer graphene is very satisfactory. The resistance of graphene synthesized by chemical vapor deposition (CVD), the acknowledged well‐suited method for growing large‐area and high quality graphene, can low to several hundreds of Ω sq^–1^ on nickel (Ni) and copper (Cu).^7^ For the graphene prepared by another common used methods, chemical and thermal methods, the sheet resistance of reduced graphene oxide (rGO) can reduce to ≈640 Ω sq^–1^ after thermal annealing at 1000 °C.^8^ When used in practical applications, these graphene films exhibited better performance than other metal conductive materials and carbon materials. However, the obtained graphene films in the experiments did not achieve what it set out to, which caused by the imperfect synthesized methods and the complicated post‐treatments. Thus various improving treatments such as heterogeneous doping and hybrid with other materials have been adopted and a great enhancement has been achieved. The extremely high carrier mobility and low sheet resistance ensure graphene a rapid transmission of electrical signals, and thus enable it a promising material for its human‐like applications.

**Figure 1:**
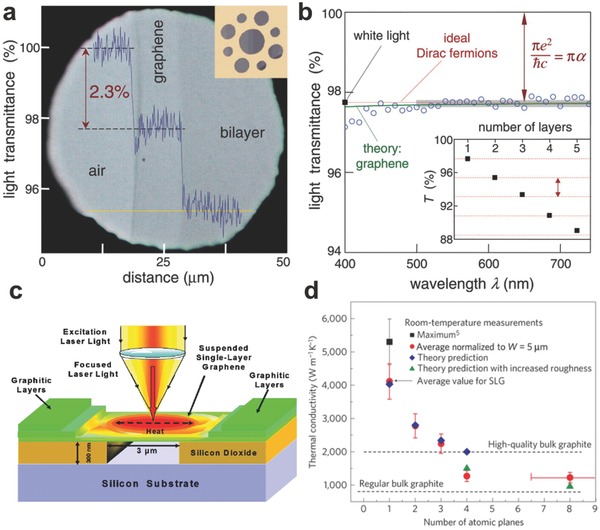
The superior properties of graphene. a–b) The transparency of graphene with different thickness. Reproduced with permission.^4^ Copyright 2008, American Association for the Advancement of Science. c) The schematic diagram of the measurement of thermal conductivity. Reproduced with permission.^13^ Copyright 2008, American Chemical Society. d) Thermal conductivity as a function of the layer number of graphene. Reproduced with permission.^14^ Copyright 2010, Nature Publishing Group.

Transparency is desired both in sense human organ and senseless object in our life. For instance, retina is inlayer of the wall of the eyeball, consisting of pigment epithelium layer and sensory layer, which is transparent. For the graphene, it has been characterized to absorb a significant of 2.3% incident white light owing to its only one atom thick.^4^ And the most important is that the transparency of graphene in near infrared region can still remain at the same level as in the visible region, while the transparency of indium tin oxides (ITO) and fluorine‐doped tin oxide (FTO) would decrease dramatically. This property of graphene makes it meet the need for many specific applications. In addition, the optical transparency of graphene is influenced by its thickness. By measuring graphene with different layer number, the opacity is found to increase 2.3% with adding each graphene layer (**Figure** **1**a, b). Thus, the controlling of the layer number appears very significant. Except for the thickness, the quality is another factor affecting the transparency of graphene. The graphene thermally reduced from graphene oxide (GO) with higher annealing temperature has a higher quality, showing higher transmittance.^9^ And the GO with lower concentration showed higher transmittance, confirming that the thickness of GO film also has the same effect on its transparency.^9^ Most of all, this prominent transmittance of graphene makes it permeable to light, just like the cornea of the human eye.

When we touch something, we can find that our skin would deform to a certain extent. And our skin can also be stretched and twist, which provide the possibility for the many activities of human beings. This is attributed to the excellent mechanical properties of shin, which is also found in graphene. Since carbon (C) atoms adopt a trigonal covalent bonding scheme of sp^2^ hybridization, the graphene also has excellent mechanical properties, such as tensile strength, shear strength and hardness. By nanoindentation in an atomic force microscope, the free‐standing monolayer graphene membrane presented that its Young's modulus is about 1.0 TPa and its fracture strength is of 130 GPa, which established that graphene is the strongest material ever measured.^10^ And these outstanding mechanical properties make it rather flexible for further applications, for instance, wearable devices. However, these values were measured with the defect‐free graphene. The quality of macroscopic graphene film is still restricted by the defects and grain boundaries. For graphene synthesized by solution‐processed method, the monolayer GO sheet had a lower Young's modulus of 207.6 ± 23.4 GPa.^11^ In addition, the average elastic modulus of GO papers self‐assembled from GO sheets was only ≈32 GPa,^12^ which should be further elevated in order to meet the needs of the applications of electronic skin and large‐area flexible touch screen.

Owning to the effective thermal transmission, humans can become warm rapidly to protect the rest of the organs when meeting a heat source in frigid weather. Relatively speaking, the rapid thermal transfer in graphene is also important for its various applications. Compared with other carbon allotropes, such as amorphous carbon (≈0.01 W mK^–1^), diamond (≈2000 W mK^–1^) and carbon nanotubes (CNTs) (≈3500 W mK^–1^), graphene has the optimal thermal conductivity in the range from (4.84 ± 0.44) × 10^3^ to (5.30 ± 0.48) × 10^3^ W mK^–1^, which offers tremendous potential for graphene as transparent heating films. In Figure 1c, a suspended monolayer graphene was first employed to measure its thermal conductivity by the excitation laser method.^13^ Similar with the transparency of graphene, its thermal conductivity also decreased with the increase of the layer numbers. The thermal conductivity in room temperature changes from ≈2800 to ≈1300 W mK^–1^ as the layer number of graphene increasing from 2 to 4, caused by the interlayer coupling of the low‐energy phonons (Figure 1d).^14^ On the other hand, graphene has a lower convective heat‐transfer coefficient than CNTs and metals (e.g., chromium), benefitting from its ideal flat surface which would determine higher final temperature and a faster heating rate.^15^

Various excellent properties of graphene indicate a very promise future. However, the reality is always cruel. The actual behavior of graphene characteristics mentioned above is not entirely as desired, due by the preparation and post‐processing, which should be further improved for the prospective applications.

### 2.2 Preparation

#### 2.2.1 Synthesis Methods

To better utilize the graphene films for the application with human‐like senses, it is necessary to synthesis graphene with extraordinary transmittance, low sheet resistance and uniformity. Such as the retina of the eye when sensing light, human‐like graphene‐based films need to be transparent. And like the human skin, it is crucial for the graphene‐based films to be flexible and relatively sensitive and extensible when sense to pressure which could then be employed in the piezoresistive sensors^16^ and wearable electronics.^17^ There are two mainstream approaches to achieve the target for various human‐like senses, CVD and solution processed method.

*CVD growth of graphene films*: Until now, many efforts have been made to synthesize homogeneous graphene films with CVD method on metal substrates. Kong et al. reported 1 to ≈12 graphene layers simultaneously obtained on polycrystalline Ni film via ambient pressure chemical vapor deposition (APCVD).^18^ Cu foil with low carbon solubility was also most commonly used to obtain predominantly single‐layer graphene.^7^, ^19^ In addition, Ni–molybdenum (Mo) alloy,^20^ platinum (Pt),^21^ cobalt (Co)^22^ also have been designed for large area monolayer graphene.

**Figure 2:**
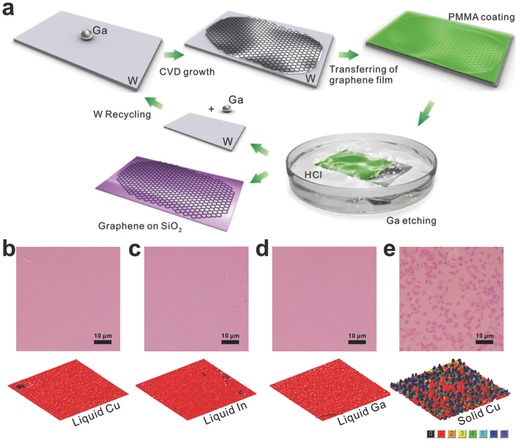
a) Schematic illustration of graphene growth over liquid Ga supported on a W substrate. Reproduced with permission.^24^ Copyright 2013, Nature Publishing Group. b–e) Typical growth results on liquid or solid metal substrates.Reproduced with permission.^23^ Copyright 2014, American Chemical Society.

It is worth stressing that, we break a new path to grow graphene film on liquid metal substrates, which is in full swing in recent years.^23^, ^24^ With the great advantages of liquid metal, such as atomic smooth and ultra‐homogeneous, we demonstrate its immense potential in growing uniform graphene with controllable layer number and crystal size. As shown in **Figure** **2**a, a droplet of gallium (Ga) was placed on a tungsten (W) foil.^24^ After a facile CVD route, via etching the Ga film in dilute hydrochloric acid, the graphene film was transferred onto a SiO_2_/Si substrate. The electron mobility of obtained single‐layer graphene reached up to 7400 cm^2^ V^–1^ s^–1^. To further understand the advantage of liquid metal substrates, we also learned the growth behavior of graphene on liquid Cu and indium (In), and it indeed revealed the superiority to solid metals, the optical microscope images were shown in Figure 2b–e.^23^ In the view of optical characteristics (Figure 2e), graphene grown on solid copper foil presented poor uniformity. Layer distribution is determined by RGB color analysis of the corresponding optical microscope images; Figure 2b−e respectively demonstrate the highly uniformity of the strictly monolayer graphene.

Besides the monolayer graphene, the production of graphene films with low sheet resistance and vast area should also be taken into account urgently. Iijima et al. successfully obtained 30‐inch graphene films with 97.4% optical transmittance and 125 Ω sq^–1^ sheet resistances, as the result of the half‐integer quantum Hall effect.^7^ After that, Hobara et al. synthesized graphene on a suspended Cu foil at a low pressure thermal CVD. Notably, a 100 m‐long transparent conductive film with a sheet resistance as low as 150 Ω sq^–1^ was obtained.^25^ As a result, choosing appropriate metal catalyst and improving growth parameters are pivotal to synthesize uniform, square meters and high conductivity graphene films with a CVD method on metal substrates, especially in SC, touch‐based electronic devices.

**Figure 3:**
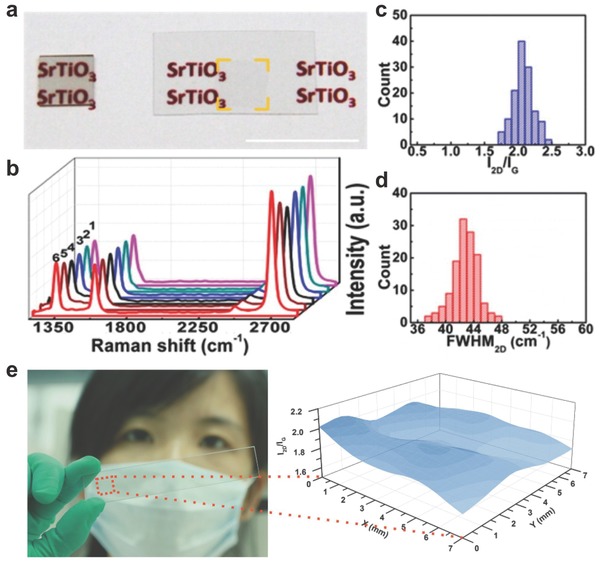
a) Photograph of a graphene/SrTiO_3_ sample (left) and a transferred graphene film to a PET sheet (right). Scale bar: 1 cm. b−d) Plot of six typically collected spectra (b), histograms of the *I*_2D_/*I*_G_ ratios (c), and fwhm_2D_ distribution (d) over a 3 × 3 mm^2^ sample area. e) Schematic diagram of the oxygen‐aided CVD growth of graphene on a SiO_2_/Si substrate. a–d) Reproduced with permission.^35^ Copyright 2014, American Chemical Society. e) Reproduced with permission.^36^

For electrical devices, a modest route for direct CVD growth of continuous and uniform graphene films are key prerequisites. What we cannot ignore is that graphene grown on metal substrates always need to be transferred on to the target substrates. This process would bring contamination, wrinkles and cracks to the graphene film.^26^, ^27^ To synthesize such films, the compatible target substrates in transparent conductors are silica,^28^, ^29^, ^30^ quartz,^31^, ^32^ sapphire,^33^ and other insulated transparent substrates, such as solid glasses^34^ and high‐*κ* SrTiO_3_ substrates.^35^ Chiu at al. used Cu foils to provide subliming Cu atoms instead of using as metal substrates.^28^ The floating Cu and H atoms could provide a remote catalyzation and a lower activation energy for pyrolysis of the CH_4_. And a monolayer graphene with carrier motilities of ≈870–1050 cm^2^ V^−1^ s^−1^ could be synthesized on single crystal SrTiO_3_.^35^ The photograph showed its uniformity and light transparency (**Figure** **3**a). In order to examine the 2D homogeneity and quality of monolayer graphene, six randomly collected spectra (Figure 3b), the plot of a histogram of the *I*_2D_/*I*_G_ ratio (Figure 3c), as well as a histogram of the FWHM_2D_ distribution (Figure 3d) were collected from 130 points over a 3 × 3 mm^2^ area. Liu et al. deposited CH_4_ with oxygen‐assisted to synthesis high‐quality polycrystalline graphene on SiO_2_ substrates.^29^ In the meantime, we first presented an approach of Ga vapor‐assisted CVD to direct graphitization of carbon fragments on quartz surfaces.^36^ By the means of this method, we first designed the defogger based on a continuous, low‐defect and large‐area monolayer graphene film (Figure 3e).

In general, direct CVD synthesis is a straightforward approach to fabricate graphene films with less contamination and continuous surface. However, for the CVD process, the cost, scale‐up issue and complex operation constraint its range commercialization. Therefore, researchers growing graphene via CVD focus more on fundamental research.

*Solution Processed Films*: Currently, liquid‐phase exfoliation and oxidation‐reduction process are the two common methods to obtain solution processed graphene films. Different from the CVD method, the solution processed method always introduced more oxygen‐containing functional groups and defects into graphene films, which is attractive to the adsorption of gas sensors and biosensors. Just like the selective and quick smell of human, functionalized graphene films show selectivity in some sensor devices.

**Figure 4:**
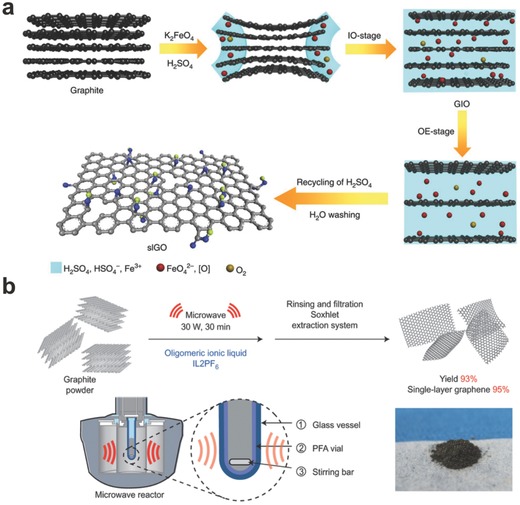
a) Schematic diagram of iron‐based environmental strategy for the fabrication of monolayer GO. Reproduced with permission.^41^ Copyright 2015, Nature Publishing Group. b) Schematic illustration of microwave‐assisted liquid‐phase exfoliation of graphite in ionic liquids. Reproduced with permission.^46^ Copyright 2015, Nature Publishing Group.

In brief, oxidation‐reduction processed is the oxidation of graphite. Since a KClO_3_‐based method was developed for the first time by Brodie et al. in 1859,^37^ it was successively modified by Staudenmaier et al.^38^ and Hofmann et al.^39^ Hummers prepared graphitic oxide from graphite.^40^ But these time‐consuming methods might bring inevitable pollution. Recently, Gao et al. reported an iron‐based environmental strategy to prepare single‐layer GO in 1h using a strong oxidant K_2_FeO_4_ (**Figure** **4**a).^41^ And this extremely powerful and ultrafast exfoliation benefit from the excellent oxidation capabilities of both K_2_FeO_4_ and the in situ generated atomic oxygen, as well as the exfoliation ability of oxygen gas. Such a green, safe, high‐efficiency, and ultralow‐cost route foreshadows the large‐scale commercial applications of graphene.

In addition, GO also is generally synthesized by the ultrasonic exfoliation of graphite oxide.^42^ By simple sonication and stirring in the water or organic solvent, graphite oxide can be completely exfoliated to produce aqueous colloidal suspensions of GO sheets. Regardless of its high potential in extendibility and applicability, liquid‐phase exfoliation has suffered from low efficacy and low product quality.^43^ It is reported that graphite and graphene,^44^ as well as ionic liquids,^45^ are thought to efficiently absorb microwaves. As shown in Figure 4b, Aida et al. reported that with the assistance of microwave irradiation, graphite suspended in molecularly engineered oligomeric ionic liquids allows for ultrahigh‐efficiency exfoliation (93% yield) with a high selectivity (95%) towards “single‐layer” graphene (thicknesses <1 nm) in 30 minutes.^46^

Whatever, high throughput solution process enables it to prepare graphene‐based films using this low cost technique. But it might decrease the transparency and sheet resistance of graphene. And eco‐friendlier and simpler solution routes are needed to investigate for ever‐growing graphene‐based devices.

#### 2.2.2 Post Treatment

*Transfer and Film Formation*: To better develop human‐like senses of graphene film, it is usually required for graphene films to be transferred to target substrates or undergo an additional film forming process. In the past years, various transfer routes have been reported,^7^, ^26^, ^47^, ^48^ which have achieved big breakthrough in many aspects, such as the decrease of cracks and wrinkles and the area expanding of graphene. The ordinary widely used transfer approach is to etch the metal substrate with the graphene supported by polymer, such as poly(methyl methacrylate) (PMMA).^49^ Owing to the variety of cracks and wrinkles appears in this typical approach, Ruoff et al. reported an improved transfer process to decrease the cracks of graphene by redeposit a second PMMA coating after the PMMA/graphene was transferred to the SiO_2_/Si substrate.^26^ This would lead the PMMA tending to relax the underlying graphene, which made the graphene contacted the substrate better.

**Figure 5:**
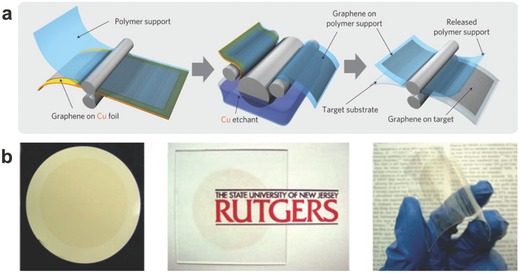
a) Roll‐to‐roll transfer of 30‐inch graphene films grown on Cu foils. Reproduced with permission.^7^ Copyright 2010, Nature Publishing Group. b) The large‐area GO membrane on different substrates. Reproduced with permission.^5^ Copyright 2008, Nature Publishing Group.

In addition, Iijima et al. invented a roll‐to‐roll transfer approach.^7^ By this method, 30‐inch monolayer graphene film has been obtained. **Figure** **5**a shows the schematic process of this roll‐based production. With a polymer support (e.g., thermal release tapes) adhered to the graphene on Cu foil, the Cu foil was etched via electrochemical process using an aqueous 0.1 m ammonium per sulphate solution (NH_4_)_2_S_2_O_8_. After that the graphene film was transferred onto the target substrate (e.g., polyethylene glycol terephthalate (PET)) by removing the adhesive force. This method successfully achieves to transfer the large‐area graphene film, which affording new opportunity for touch devices. However, such graphene films cannot avoid cracks and folds. In order to obtain large‐area graphene without cracks, Loh et al. developed a face‐to‐face transfer method for the graphene films in wafer‐scale, which relied on the gas bubbles and capillary bridges between the underlying substrate and the graphene film during etching of the metal catalyst.^47^ This method is operable to batch processing which would accelerate the applications of graphene.

Inspired by the natural phenomenon of a snail secreting mucus, we developed a novel sliding transfer strategy recently.^50^ When facing with the target substrate, with a horizontal sliding force, the graphene film on the original liquid (melted) substrate can completely adhere to the target substrate, ultrafast and highly controllable. While for sliding transfer, most importantly, no pollution sources were introduced into the graphene film.

Until now, significant advances have been achieved during the past years. However, more endeavor still needed to be focused on the larger‐area uniform graphene films with less cracks and wrinkles.

Different from the graphene grown by CVD, GO sheets always have been heavily oxygenated, with carbonyl and carbonxyl groups located at the sheet edges, as well as hydroxyl and epoxide functional groups on the basal planes.^51^ For solution‐processed graphene, the large number of GO flakes should be dispersed in water or organic solvents before forming a transparent thin film. Compared with the transfer of graphene film, the film‐formation of GO demonstrates to be more convenient and scale‐up, as well as no limit to substrates. Numerous approaches have been adopted to prepare GO films, including drop‐casting,^52^, ^53^ spin‐coating,^54^ vacuum filtration,^5^, ^55^ or other assembly processes^56^, ^57^, ^58^, ^59^ and so on.

Chen and Bao et al. spin‐coated GO dispersions on the prepared substrate and controlled its thickness by adjusting the rotating speed.^54^ With low rotating speed to uniformly spread and high speed to a thin layer, they found that the single‐layered graphene films shown sheet resistances as low as ≈10^2^–10^3^ Ω sq^–1^ and with 80% transmittance for 550 nm light. Under different rotating speed, all GO thin films reveal to be with high electrical conductivity and transparency in varying degrees. This route, as well as drop‐casting, is still in widespread use today.

It's worth mentioning that besides drop‐casting and spin‐coating, vacuum filtration is also an alternative solution for large‐area continuous graphene films. As Chhowalla's group described, by the means of a commercial mixed cellulose ester membrane with an average pore size of 25 nm, they easily realize the vacuum filtration of a GO suspension.^5^ By way of placing the membrane with the film‐side faced down, the GO flakes on the filter membrane can be transferred onto various substrates, for instance, glass and plastic (Figure 5b). This technique tremendously boosts the application of graphene in scale‐up and large‐area flexible devices, such as electronic skin.

In short, towards the high‐quality graphene grown on metal substrate, it is inevitable to etch the metal and transfer the film to target substrates. However, the transfer process is still confronted with the mishap of the cracks and wrinkles on large area graphene film. To the solution processed GO films, the functional groups in some extent sow the seeds for adsorption of various chemical molecules.

*Optimization*: As is known to all, the neurons in human nervous system have special structures, allowing them to send signals precisely and rapidly to other cells. To utilize the graphene film in human‐like sense devices, the promotion of conductivity which is similar to electrical signal transmission is crucial. Although the conductivity of graphene in theory gives it a promising future, the reality does not match with the theory due to various factors caused by the defects and cracks from synthesis and post treatment process. Therefore, chemical heterogeneous doping is a useful method to improve the graphene sheet resistance. SOCl_2_,^60^, ^61^ SOBr_2_,^62^ HNO_3_,^7^, ^60^, ^62^, ^63^, ^64^ and AuCl_3_ 64, 65 were commonly used as chemical dopants. The chemical dopants were always dissolved in a solvent and then spin‐coated onto the graphene film on the target substrate.

**Figure 6:**
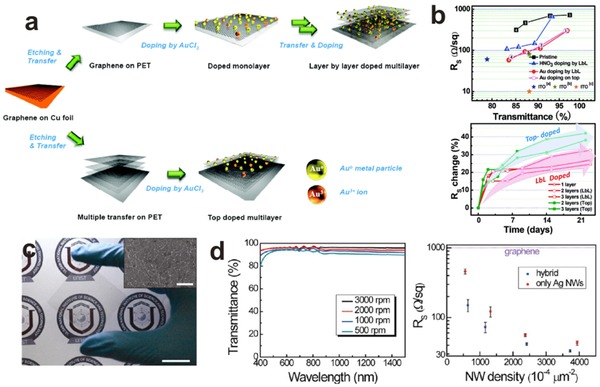
a) Schematic of the layer‐by‐layer doping strategy. b) The sheet resistance and transmittance of LbL‐doped graphene film in various conditions. c) Photograph of graphene–AgNW hybrid film with a PET substrate. The inset shows a SEM image of this hybrid. d) Optical transmittance spectra and the sheet resistances of the hybrid film. a,b) Reproduced with permission.^66^ Copyright 2010, American Chemical Society. c,d) Reproduced with permission.^70^ Copyright 2013, American Chemical Society.

AuCl_3_ chemical doping was the most commonly used way to enhance the conductivity of graphene. When dissolved in a solvent, the ionic conformation in AuCl_3_ was affected by the amount of coordinating agent. Kong et al. chose nitromethane as solvent of AuCl_3_.^65^ By optimizing the AuCl_3_ doping concentration, the sheet resistance of the graphene film can be decreased to 150 Ω sq^–1^ with a transmittance of 87%, which can be comparable to the ITO films. However, the instability of the AuCl_3_ doping would dramatically increase the sheet resistance in the practical applications. To alleviate this problem, Lee et al. proposed a novel layer‐by‐layer (LbL) doping method (**Figure** **6**a).^66^ Briefly, after a graphene film was removed onto the target substrate, AuCl_3_ solution was spin‐coated on the graphene film. And then, this LBL process was repeated for several times. The sheet resistance of the LbL‐doped four‐layer graphene film can be decreased to 54 Ω sq^–1^ at transmittance of 85% (Figure 6b).

The instability of graphene‐dopant coactions in the air or under thermal loading limits its lifetime and thus causes an increase of sheet resistance (*R*_s_).^67^ Hence, beyond chemical doping mentioned above, hybrid structures, e.g., introducing CNTs^68^, ^69^ or metal nanostructures^70^, ^71^, ^72^, ^73^ also have been developed to solve this problem in recent years.

Lee and co‐workers spin‐coated a thin layer of Ag nanowires (AgNWs) network on the top of the graphene film.^70^ Figure 6c is the image of graphene–AgNWs hybrid film on a PET substrate, while the insect is a scanning electron microscopy (SEM) image. In Figure 6d, Lee et al. also made a comparison of film transparency under various rotating speed. This hybrid structure could reduce R_s_ down to ≈33 Ω sq^–1^ with a transparency of 94%, while retaining the electrical and optical properties reliably opposing the thermal oxidation conditions, and also present superb mechanical flexibility and stretchability.

Recently, Cao et al. developed a blown bubble method which could directly fabricate CNT–graphene hybrid films.^68^ They used PMMA combined with CNT arrays as the polymer matrix to blow bubbles. After an annealing treatment, the PMMA were then transformed in graphene. The hybrid films exhibited enhanced conductivity and structural integrality.

In summary, two optimization strategies both could improve the conductivity of graphene films. The chemical doping method can decrease the *R*_s_ by augmenting carrier density, while the graphene‐based hybrid materials can provide more conductive pathways, leading to locally increase in conductivity.

## 3 The Human‐Like Senses and Feedbacks

### 3.1 The Human‐Like Senses

#### 3.1.1 Vision—Light Sensing

**Figure 7:**
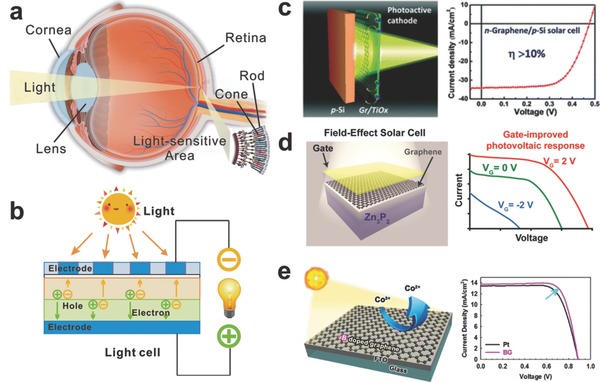
a) Schematic diagram of the human eye light sensing. b) Schematic diagram of the SC light sensing. c) Schematic image of a TiO_x_/graphene/p‐Si Schottky junction SC and its performance with and without PMMA antireflective coating. d) Schematic image of a graphene–Zn_3_P_2_ SC and its photovoltaic response. e) Schematic image of a B‐doped graphene SC and its current–voltage characteristics. a–c) Reproduced with permission.^79^ Copyright 2015, Royal Society of Chemistry. d) Reproduced with permission.^81^ Copyright 2014, American Chemical Society. e) Reproduced with permission.^82^ Copyright 2014, American Chemical Society.

As we all know, through the eyes, human perceive and assimilate information from the external world. Light entering the eye is refracted when it passes through the cornea, as the **Figure** **7**a shows. After that, the lens of the eye concentrates an image of its surroundings onto the retina which is a light‐sensitive membrane in the back of the eye. The retina is an isolated part of the brain, serving as a transducer which converts light patterns into neuronal signals. Similarly, a SC, working as the human eye, is a device that converts light signals directly into electrical signals (Figure 7b), with varying electrical characteristics when exposed to light, such as current, voltage, or resistance.

The SCs has long been recognized as a clean energy technology which utilizes the planet's most abundant and widespread renewable energy source—the sun. The technology directly converts sunlight to electricity without any moving parts or environmental emissions during operating process. As a key component of SC, the transparent electrode serves the dual functions: transmittance and electroconductibility, whereas the ITO is most commonly used. And yet, ITO has inevitable disadvantages, such as chemical and thermal instability, high production cost and friable. Driven by the progress in technology and manufacturing scale, more requests have been made on low cost and flexible SCs, which is a territory most semiconductor giants are looking forward to develop but the commercial ITO can hardly achieve. More attractive than ITO, graphene has been incorporated into each aspect of SC due to its extremely high transmittance (97.7%),^4^ low sheet resistance (30 Ω sq^–1^) and flexibility^5^ in theory. Here, we will summarize the contributions of graphene in several kinds of common SCs.

By virtue of the superior power conversion efficiencies (PCEs), easy preparation, as well as low cost, perovskite SCs with a perovskite‐structured material as the active layer have aroused considerable attention in recent years.^74^, ^75^ Even so, the utilization of graphene film in perovskite SCs has been rarely explored up to now, therein graphene‐based films have been served as interlayers with success for high‐efficiency charge transfer or collecting.^76^, ^77^ Recently, Yan et al. first reported the laminating stacked multilayer graphene serving as top electrode for semitransparent perovskite SC.^78^ They dramatically improved the conductivity of graphene with a thin layer of poly (3,4‐ethylenedioxythiophene): poly(styrenesulfonate) (PEDOT:PSS) which was adhered to the perovskite active layer during the lamination process. When illuminated from the bottom (FTO side) and the top (graphene side) electrodes, the SCs show an average PCEs reaching up to 12.02% and 11.65%.

Multi‐junction (MJ) SCs are those SCs with multiple p–n junctions which produce an electric current reacting to the light with different wavelengths. To absorb a broader range of wavelengths and then significantly improve the cell's sunlight to PCEs, Chen et al. reported a novel “sunlight‐activated” graphene‐heterostructure film with a strong light‐matter interaction at the interfaces.^79^ The photoactive graphene/TiO*_x_*‐heterostructure transparent cathode was utilized to prepare an n‐graphene/p‐Si Schottky junction SC as shown in Figure 7c, achieving a record‐high PCE (>10%). Moreover, this graphene‐based film shows a high transparency of approximately 96.0% at 550 nm, which is comparable to that of the monolayer graphene.

In 2012, Regan et al. demonstrated field‐effect SCs, using a transparent top gate to regulate the potential barrier and the electric field in a metal‐semiconductor junction for the purpose of separating photo generated electron‐hole pairs.^80^ Zettl et al. presented a new type of field‐effect SC utilizing graphene and zinc phosphide (Zn_3_P_2_) to form a tunable junction barrier thin‐film light absorber.^81^ To control the junction barrier between the graphene and a semiconductor, the Fermi level tuning of graphene is the key point. As it shows in Figure 7d, the results demonstrated that the modulation of graphene‐semiconductor junction renders its performance by improving parameters. The 1.9% efficiency managed in this work is acceptable but has significant room for improvement.

Although the conversion efficiency is less than that of the best thin‐film SCs, dye‐sensitized solar cells (DSSCs) can be designed into flexible sheets whose price/performance ratio are high enough to compare favorably with fossil fuel electrical generation. As it discussed in the previous section, doping heteroatoms into a graphitic carbon framework is an efficient way to activate graphene and to enhance its conductivity. Baek et al. reported that boron‐doped (B‐doped) graphene (BG) is prepared and tested as a counter electrode (CE) in DSSCs in conjunction with Co(bpy)_3_^2+/3+^ redox couple.^82^ As a result, the DSSC fabricated with BG CE exhibits superior PCE (9.21%) to that of DSSC with Pt CE (8.45%) in Figure 7e. Also doping heteroatoms, Qiu et al. developed an efficient strategy to fabricate nitrogen‐doped (N‐doped) graphene via chemical unzipping of CNTs coupled via a nitrogen doping process.^83^ Their work has profound understood the characteristic role of N species on graphene for the triiodide reduction, and afforded an efficient strategy for fabricating high‐efficiency graphene‐based electrodes with surface enriched active sites.

As another type of human‐like light‐sensitive device, graphene‐based PD has received an enormous amount of attention due to the wavelength‐independent light absorption and high operating band width of graphene.^84^, ^85^, ^86^ Furthermore, the high carrier mobility enables graphene an ultrafast transformation of photons or plasmons to electrical currents or voltages.^87^, ^88^ Although the absorption spectrum of graphene covers the entire ultraviolet to far‐infrared range,^4^, ^89^ the photoresponsivity of graphene‐based PD is limited by small optical absorption of a monolayer of carbon atoms. Hence, the current research focuses more on improving the photoresponsivity of the graphene‐based PD while remaining its excellent broadband.

Zhong et al. reported an ultra‐broadband PD design based on a graphene double‐layer heterostructure, demonstrating a mid‐infrared responsivity higher than 1 A W^–1^ which is suitable for most applications.^86^ This detector is a phototransistor composed by a pair of stacked graphene monolayers sandwiching a thin tunnel barrier. In contrast to conventional phototransistors, when under optical illumination, the photoexcited hot carriers came about from the top graphene layer tunnel into the bottom layer at room temperature, leading to a charge accumulation on the gate and a strong photogating effect on the channel conductance. And this sandwich‐like graphene‐based PD is comparable with state‐of‐the‐art infrared PDs operating at low temperature.^90^, ^91^ Of course, other measures also have been adopted to improve the photoresponsivity. Li et al. developed a graphene–carbon nanotube hybrid film with a photoconductive gain of ≈10^5^ electrons per photon.^85^ The broadband PD (covering 400–1550 nm) based on such hybrid films exhibits a high photoresponsivity of > 100 A W^–1^ and a fast response time of ≈100 μs. Analogous to this, researchers also introduced other additives into the graphene‐based PD for improving the responsivity, for instance, boron nitride^92^ and gold oxide.^93^

In summary, several strategies have been adopted to enhance the PCE of the SCs and the photoresponsivity of the PD via the appropriate modification of graphene films. Corresponding to the transparency and nerve impulse of light‐sensitive human eye, the transmittance and carrier dynamics are essential for the SCs of graphene films, which have made rapid progress.

#### 3.1.2 Hearing—Sound Sensing

**Figure 8:**
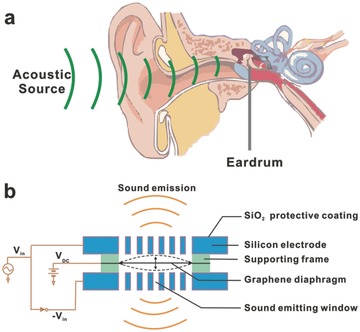
a) Structure diagram of human hearing system. b) Schematic diagram of SEDs.

Hearing, or auditory perception, perceives the sound by sensing vibrations, pressure variation of the surrounding medium with time, via an organ such as the ear. The eardrum would detect the vibrations, also known as mechanical waves, and then transduce them into nerve impulses. The human ear consists of three main components: the outer ear, the middle ear, and the inner ear (**Figure** **8**a). The outer ear is composed of the pinna, the visible part of the ear, and the ear canal, which terminates at the eardrum. The sound waves toward the eardrum are focused by pinna through the ear canal. Here, the eardrum is an airtight thin membrane, which vibrates following the waveform of the sound waves arrived there. Transmitting the sound from the air to the ossicles, and then the oval window in the fluid‐filled cochlea, the eardrum ultimately converts and amplifies vibration in air to vibration in the fluid. based on this theory, people developed amounts of sound‐emitting devices (SEDs), such as an earphone, a loudspeaker, and a microphone. Figure 8b is the schematic diagram of SEDs. Based on the membrane vibrations, these devices can produce a sound with a frequency response to its resonance peaks. And the common ground of these devices is a vibration sensitive membrane that working as an eardrum.

In 2011, Ren et al. first patterned graphene on paper substrates and demonstrated that graphene can emit sound.^94^ It was found that graphene has a significant flat frequency response in the wide ultrasound range ≈20–50 kHz. Additionally, graphene exhibits ultra‐small heat capacity per unit area (HCPUA), more efficiently converting joule heating to sound waves.^94^, ^95^, ^96^, ^97^, ^98^, ^99^, ^100^ It has been also reported that single‐layer graphene (SLG) presents the lowest HCPUA,^101^ demonstrating more efficient than other materials.

**Figure 9:**
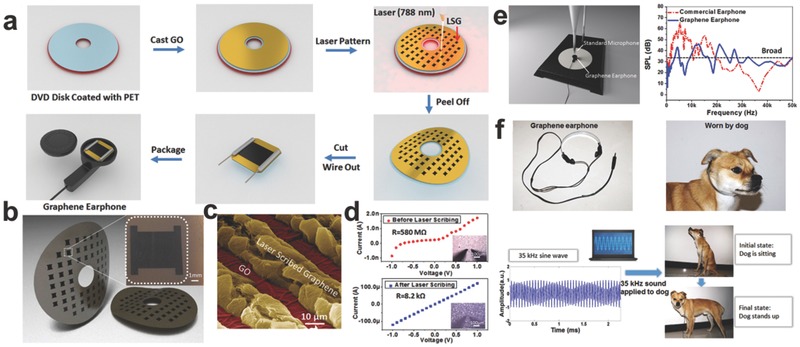
a) Fabrication process of the graphene earphone. b) Flexible graphene earphones in wafer‐scale. c) Color rendered SEM image of the LSG and GO. d) Electrical properties before and after laser scribing. e) Experimental setup and frequency characteristics of the graphene earphone. f) The testing of graphene earphones on the dog. Reproduced with permission.^98^ Copyright 2011, American Chemical Society.

Since then, Ren et al. developed a high‐efficiency route for the fabrication of real graphene earphones using laser‐scribed graphene (LSG).^98^ As is shown in **Figure** **9**a, the ultrafast graphene growth was obtained at precise locations by a mask‐free and programmable laser scribing technology. By laser‐induce, the GO film applied on a polyethylene terephthalate (PET) film‐coated DVD media disk was then reduced into graphene. The morphology and structure of graphene sheets are also shown in Figure 9b, c. And Figure 9d shows the electrical properties of graphene before and after laser scribing. In Figure 9e, the graphene earphone presents a predictable flat frequency ranging from 100 Hz to 50 kHz. Compared with commercial earphones, the graphene earphone also has a lower sound pressure fluctuation. More interestingly, this graphene earphone can be applied on a previously trained dog, as shown in Figure 9f. According to the hearing range of dogs is within 30 Hz to 60 kHz, the dog has subjected to the training of standing up after hearing a 35 kHz sine wave. As a result, the dog was first ordered to sit down and then stood up as expected when a 35 kHz sine wave was played to it. Recently, Ren's group demonstrated another flexible, transparent and ultrathin single‐layer graphene (SLG) earphone.^97^ The SLG was grown on Cu at 1000 ºC by means of a CVD route. Compared with a conventional earphone, this new type of earphone operates in a broader ranging from 20 Hz to 200 kHz, with a highest sound pressure level of 70 dB, showing a broader frequency response and a lower fluctuation. Moreover, the SLG earphone technology bids fair to bring transparent flexible earphone in the acoustic field.

Zettl et al. presented a graphene‐based wideband microphone and a correlative ultrasonic radio that are expected to be used for wireless communication.^95^ As a result, from the audible region (≈20 Hz–20 kHz) to the ultrasonic region (20 kHz to at least 0.5 MHz), the graphene‐based acoustic transmitters and receivers still functions well even in such a rather wide bandwidth. The receiver component has been independently field‐tested in recording wild bat calls. It demonstrated that an amplitude and frequency‐modulated and electroacoustic range‐finding method is established with the ultrasonic radio with submillimeter accuracy.

**Figure 10:**
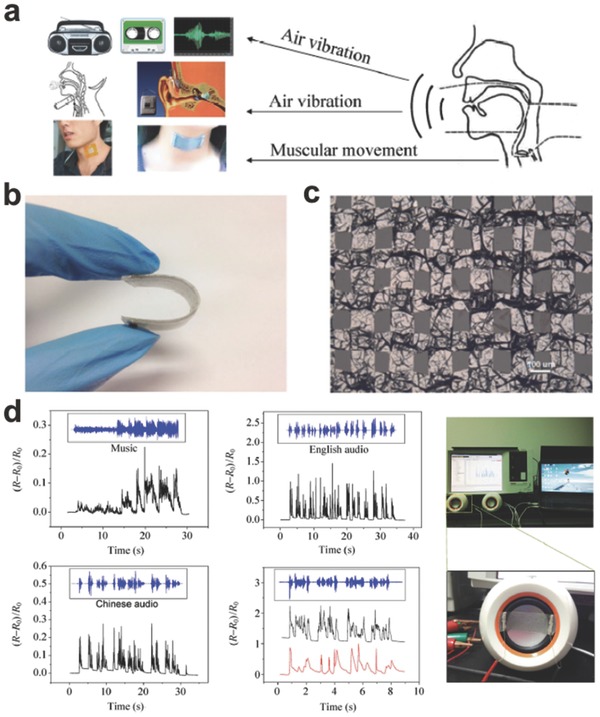
a) Three ways of collecting and recognizing human voices. b) Photograph of a bent strain sensor. c) SEM image of GWFs. d) Recognition signals compared with the sounds from the loudspeaker. Reproduced with permission.^96^ Copyright 2015, Springer.

Meanwhile, Zhu et al. fabricated a wearable and high‐precision sensor with thin films of well‐designed graphene woven fabrics (GWFs) for the sound signal acquisition and recognition.^96^ Three ways of collecting and recognizing human voices are shown in **Figure** **10**a. Figure 10b,c show a photograph and SEM image of the thin film sensor. After being stretched, the resistance would be increased arising from the generated high density of random cracks in the network. The detector exhibited ultrahigh sensitivity to tiny strains and vibrations, so as to detect sound waves even at low sampling frequencies when serving as an electronic skin covering the human throat. The audio experiments were performed by utilizing both English and Chinese music with the GWF‐based sensor on the vibrating membrane of a loudspeaker, as shown in Figure 10d. Due to its excellent flexibility, high sensitivity, good reversibility, and overall biocompatibility, the GWF‐based sensor is expected to be useful for earthquake detection and animal communication, as well as the robotic voice development.

#### 3.1.3 Olfaction—Smell Sensing

Olfaction is mediated by specialized sensory cells in our nasal cavity. For human, olfaction occurs when odorant molecules combine with the receptor, which will produce an action potential. The responses to an odorant molecule can be characterized by measuring the electro‐olfactogram or calcium imaging of receptor neuron terminals. Put the same point the other way around: can we detect the variety of gas molecules in the environment by fabricating similar devices with our olfaction receptor neuron?

**Figure 11:**
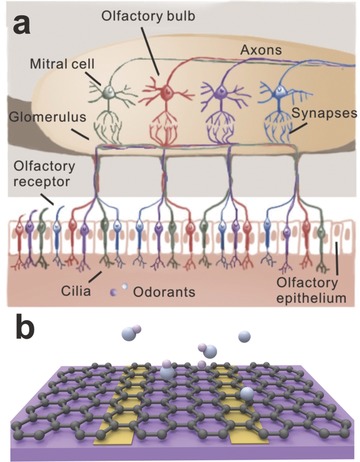
a) The schematic illustration of the mechanism of smell. Reproduced with permission.^105^ Copyright 2011, Nature Publishing Group. b) The adsorption of the gas molecules on the surface of graphene serving as gas sensor.

As one of the most anticipated materials in the future, graphene also can be used in gas sensor attributing to its large theoretical specific surface area of 2630 m^2^ g^–1^, high carrier mobility of 200 000 cm^2^ V^–1^ s^–1^, high carrier density of ≈10^12^ cm^–2^ and low resistivity of 10^–6^ Ω,^102^, ^103^, ^104^ especially the inherently low electrical noise. Similar as the binding between odor and olfaction receptor neuron (**Figure** **11**),^105^ gas molecules would adsorb on the graphene sheets and induce conductance changes of graphene, which can be detected by measuring the changes of the electrical signal of graphene. Here, there are three modes of the interaction between gas molecules with graphene: a) influencing the conductance of graphene by redistributing electrons, such as H_2_O;^106^ b) contributing electrons or holes to graphene to change its electron concentration, such as NO_2_;^107^ c) forming covalent bonds.^108^ Because of the adsorption of water molecules in air, the graphene often exhibits p‐type. Thus, the resistance of graphene would decrease as the adsorption of the electronic acceptors (e.g., NO_2_) and increase as the adsorption of the electronic donors (e.g., NH_3_).^109^

The mechanical exfoliated graphene was firstly used to fabricate graphene‐based gas sensor,^106^ which showed good sensitivity in detecting NO_2_, H_2_O, CO and NH_3_. What's more, the graphene‐based gas sensor also allowed the maximum sensitivity for detecting individual gas molecules. Furthermore, the graphene film grown by CVD methods also can be used to fabricate gas sensor. Wlodarski et al. reported that the adsorption of O_2_ molecules on monolayer CVD graphene can result to significant changes of the electrical resistance.^110^ Shi et al. also fabricated these CVD graphene‐based gas sensors which exhibited the sensitive responses to NO_2_ of 100 ppm and NH_3_ of 1%. And NH_3_ can be selectively detected from CH_4_ or H_2_, which shows an excellent selectivity.^111^

In order to improve the performances of graphene‐based gas sensors, including the sensitivity and the selectivity, functionalized graphene was used. GO with a certain amount of oxygen groups was fabricated as gas sensors after a chemical or thermal reduction process. Here the oxygen groups can act as adsorption sites, which would make the gas molecules easier to adsorb on the graphene. By forming GO continuous film on an insulating substrate from the aqueous dispersion, Sheehan et al. fabricated GO‐based gas sensors.^112^ What's more, the active oxygen defects on the surface could selectively detect different molecules. For example, HCN molecules can more strongly interact with the defective graphene than the pristine graphene. When utilizing different reductants, the selectivity of rGO‐based gas sensor is greatly improved. For rGO reduced by p‐phenylenediamine,^113^ the response to dimethyl methyl phosphonate (DMMP) of this sensor was enhanced to 4.7 times stronger than that of the sensor based on rGO, which also exhibited a better response repeatability. Besides, the rGO‐based gas sensor could also selectively detect corrosive vapors such as NO_2_ and Cl_2_ after reduced by ascorbic acid, whose limit of detection (LOD) was up to the range of 100 ppm to 500 ppb.^114^ Sulfonated rGO and ethylenediamine‐modified rGO (EDA–G) were also prepared and exhibited higher response of 4 to 16 times toward NO_2_ than the pristine rGO.^115^ However, it is regrettable that the recovery of the sensor was relatively slower than the pristine graphene due to the strong interactions of detected molecules with graphene.

Another way to achieve highly sensitive detection of various gas species is to hybrid the graphene films with other species, such as nanoparticles, polymer and so on. The hybridization of metal or metal oxide nanoparticles with graphene has demonstrated a significant synergistic effect towards gas sensing. These hybrids possess modulated electronic properties, enabling them to enhance the selectivity and sensitivity. For instance, vertically aligned ZnO nanorods was utilized to prepare hybrid material with chemically converted graphene^116^ and CVD graphene,^117^ in order to maximize the surface area to achieve a fast and easy gas transport. In addition, Cu_2_O nanowire was also used as a hybrid source to compound with rGO sheets, which have enhanced the response for 2 ppm NO_2_ from 22.5% to 67.8%.^118^ And its theoretically calculated LOD of 64 ppb was lower than the rGO and Cu_2_O, which showed a significantly enhanced sensitivity.

**Figure 12:**
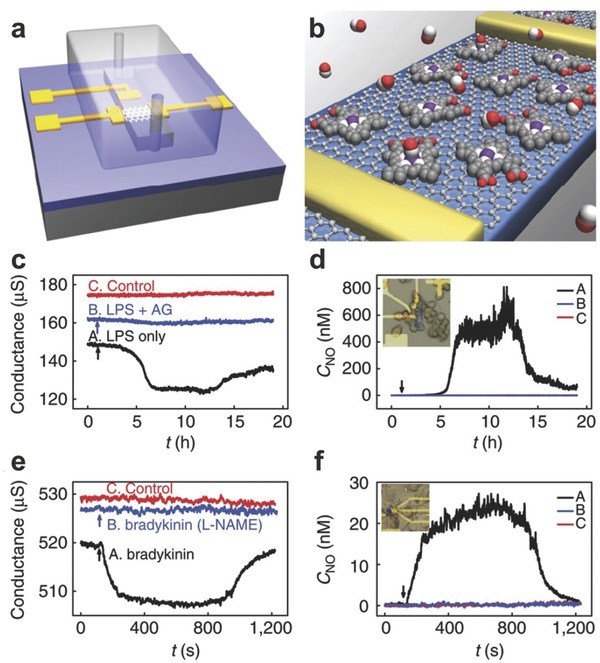
Real‐time monitoring of NO released from living cells. a–b) The schematic of graphene‐hemin gas sensor. c–d) Real‐time detection of the NO concentration released from Raw 264.7 macrophage cells. e–f) Real‐time monitoring of NO released from HUVECs. Reproduced with permission.^123^ Copyright 2013, Nature Publishing Group.

Graphene/polymer composite also can be utilized to effectively enhance the sensitivity and selectivity of graphene‐based gas sensor. PMMA was first composited to the graphene sheets to enhance carrier scattering to result in a strong and reversible electrical response to nonanal vapor in ppm level.^119^ What's important is that its signal can recover in a short time. In addition, polypyrene (PPr), polypyrrole (PPy) and polyaniline (PANI) were also utilized to form specific selective gas sensor.^120^, ^121^, ^122^ Recently, the real‐time gas sensor of NO has been successfully fabricated to detect the NO with nanomolar sensitivity.^123^ This gas sensor was based on the hemin‐functionalized graphene field effect transistors (**Figure** **12**a,b). In this work, the mechanical exfoliated graphene was functionalized by hemin that has large binding constant and high selectivity to NO. As a result, graphene‐hemin sensors of 0.25 mm^2^ were fabricated and thus a sub‐nanomolar detection limit of 0.3 nm NO was achieved. What's more, the sensitivity has also been confirmed in physiological solutions and the real‐time detection of NO released from macrophage cells (Figure 12c) and endothelial cells (Figure 12d) have successfully been carried.

**Figure 13:**
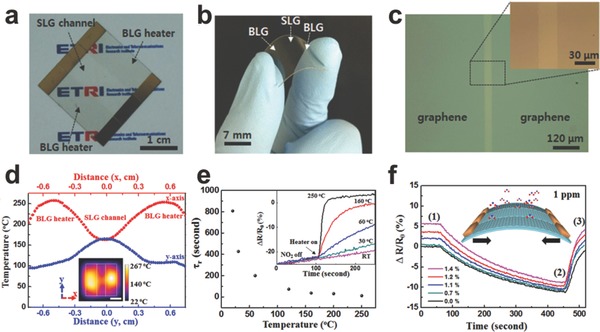
Integration of graphene gas sensor and graphene heater. a–b) Optical images of SLG sensor‐BLG heater. c) The interface between SLG sensor‐BLG heater. d) Temperature distribution of SLG sensor‐BLG heater device. e) Recovering time reducing with the increasing of temperature. f) The ΔR/R_0_ of SLG sensor as a function of time. Reproduced with permission.^107^

Graphene‐based gas sensors have exhibited extremely high sensitivity and selectivity which can achieve ultrafast response for specific gas molecules. Whereas, they always have poor reversibility, which is caused by the strong combination between adsorbates and graphene.^124^ Ultraviolet (UV) light was successfully induced to promote the desorption of gas molecules from the sensing layer.^125^ Furthermore, heating was also an effective method to recover sufficiently. By integrating with graphene sensor and graphene heater (**Figure** **13**), the faster recovering of the graphene sensor has been proved (Figure 13e).^107^ And attributing to the flexibility of graphene, the sensitivity of the sensor was secured with ΔR/R_0_ of 12% for 1 ppm NO_2_ when applied bending strain of 1.4% (Figure 13f).

#### 3.1.4 Gustation—Taste Sensing

**Figure 14:**
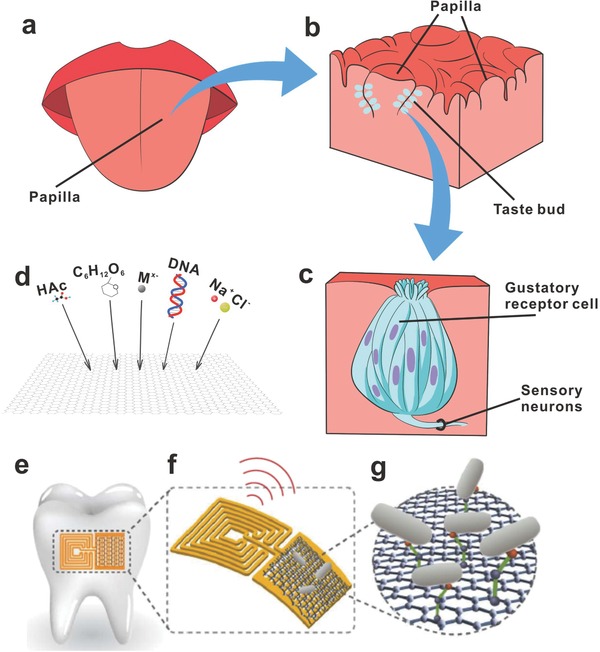
a) Dorsum of tongue showing location of papillae. b) The specific structure of papillae. c) Structure of a taste bud. d) The sensing of acetic acid, glucose, heavy metal ions, DNA and inorganic salt with graphene‐based biosensors. e) Biotransfer of the nanosensing architecture onto the surface of a tooth. f) Magnified schematic of the sensing element, illustrating wireless readout. g) Binding of pathogenic bacteria by peptides self‐assembled on the graphene nanotransducer. e–g) Reproduced with permision.^148^ Copyright 2012, Nature Publishing Group.

Of the five traditional senses, gustation is the sense generated by the stimulations of food or other substances on the tongue. It is the sensory impression produced when the substance in the mouth reacts chemically with the gustatory receptor cells. There are thousands of small bumps called papillae on the tongue (**Figure** **14**a) and hundreds of taste buds are located in each papilla (Figure 14b). Most of the taste buds are on the back side and front side of the tongue. And others are situated on the roof, corner and back side of the mouth, or in the throat. As shown in Figure 14c, each taste bud contains fifty to one hundred gustatory receptor cells.

A biosensor is like the taste bud, which functions in the same way of human gustation. The recognition layer in a biosensor will act as the “gustatory receptor cells”, which are designed to interact with the target analytes to produce a primary signal. After the primary signal was generated, it will be transferred into another easily measured and quantified signal (i.e., transduces) by the transducer element which plays a part in an electrochemical or physicochemical way. As for graphene film‐based biosensors, the final signals generally are electrical signals, and graphene will serve as a highly conducting network (just like human neural network which also functions by the transfer of electrical signals). With high specific surface area and excellent electrical conductivity, graphene works the same as or even better than the “sensory neuron” which could transfer the electrical signals rapidly and accurately.

The gustation is categorized into five elementary tastes (sweetness, sourness, saltiness, bitterness, umami). Taste buds can differentiate various different tastes through interactions with different molecules and ions. Sweetness, bitterness, and umami are induced by the binding of the molecules to G protein‐coupled receptors on the cell membranes of taste buds. Sourness and saltiness are sensed when hydrogen ions or alkali metal come into the taste buds, respectively.

Sweetness is due to sucrose, glucose, etc. The gustation of sweetness is just like the sensing of small biomolecules. Dopamine (DA), ascorbic acid (AA) and uric acid (UA) are regarded as the three kinds of small biomolecules that have important effects in physiological features of organisms. Sheng et al. investigated the sensing performance of N‐doped graphene for these molecules and got detection limits of 2.5 × 10^–7^ m, 2.2 × 10^–6^ m and 4.5 × 10^–8^ m, respectively.^126^ Ping et al. introduced a graphene‐based screen‐printing ink which could also show high resolution and sensitivity for detecting these molecules.^127^ β‐nicotinamide adenine dinucleotide (NADH) is an essential small biomolecule concerned with metabolic process. Govindhan et al. demonstrated an Au/rGO electrode to detect NADH via CV. Compared to previous works, the Au/rGO sensor had an almost 100 times higher sensitivity and extremely low detecting limit.^128^ H_2_O_2_ generates in various enzymatic reactions. Zhang et al. designed PSSA‐g‐PPY functionalized graphene to detect H_2_O_2_, which can also be used to detect hypoxanthine in fish samples.^129^ In addition, other small biomolecules such as quercetin,^130^ isoquercitrin, baicalin,^131^ cholesterol^132^ and ATP^133^ can also be detected with graphene film‐based biosensors. However, while introducing them into clinical application, the graphene film‐based biosensor system must be further improved for simplified, reasonable cost and etc.

Sourness is generated from hydrogen ions of HCl, acetic acid or citric acid. The gustation of sourness is just like the sensing of pH. pH is of great concern to all life forms. A number of biosensors based on graphene films have been utilized as pH sensors in the past few years. Among these biosensors, different approaches have been utilized to monitor pH such as SGFETs sensors, GO‐based fluorescent sensors, electrochemical sensors and chemiresistors sensors. Graphene and derivates with large π–π conjugated system itself can be hybridized with pH sensitive fluorescent molecules. Therefore, the detection of fluorescent intensity based on GO is another effective strategy for pH sensing. Hsieh et al. constructed GO‐based nanoplatforms to monitor cellular pH, which achieved both pH sensing and pH‐dependent drug delivery.^134^

Saltiness is generated mainly by NaCl while the bitterness is generated by MgCl_2_, quinine and caffeine. The gustation of saltiness is just like the sensing of metal ions. For graphene film–based biosensors, the sensing of heavy metal ions is of much importance. Being very toxic in human beings, heavy metal ions are of extreme concern. Numerous sensors of heavy metal ions based on graphene have been widely studied since their extraordinary properties were revealed. A variety of heavy metal ions (such as Ag^+^, Au^3+^, Cu^2+^, Cd^2+^, Fe^3+^, Hg^2+^, Mn^2+^ and Pb^2+^) have been well detected via these graphene‐based sensors.^135^, ^136^, ^137^, ^138^, ^139^, ^140^, ^141^, ^142^ As is reported, abundant researches on heavy metal ions sensing related to graphene have emerged in recent years. With high conductivity, large area, and π–π conjugated structures, graphene‐based biosensors have broadened the detection of the heavy metal ions to be more precise, more facile, lower cost, and environment‐friendly.

The last taste is umami, which is a Japanese term for “deliciousness”, triggered by disodium guanylate in mushrooms, monosodium glutamate in seaweeds, and disodium inosinate in meat and fish. Umami is produced by the binding of the molecules to the G protein‐coupled receptors on the surface of taste buds. Protein is one of the fundamental constituents of human bodies, also an important research target because it is the bridge between genes and physiological activities. An accurate and fast detection of proteins is also attractive to researchers. Conventional methods to detect a certain protein usually require corresponding probe molecule. Graphene here can be a substrate that provides enough active sites to immobilize these probes and enhance the electron transfer. Wang et al. introduced a kind of ionic liquid modified graphene‐based bovine hemoglobin (BHb) sensor and conduct the electrochemical determination of the target protein.^143^ In that work, they determined the optimized condition under which the functionalized graphene sensor exhibits a fast rebinding dynamics. Their sensing system showed excellent performance for BHb detection with a wide linear range from 1.0 × 10^–10^ to 1.0 × 10^–3^ g L^–1^ and a detection limit of 3.09 × 10^–11^ g L^–1^. Zhuo et al. conducted a sensing system of thrombin based on ferrocene–graphene sheets.^144^ Due to the effective quenching pattern of ferrocene to Ru (bpy)_3_^2+^ tagged thrombin, they obtained a low detection limit of 2.1 × 10^–10^ m. Afterwards, Xue et al. constructed a more effective sensor for thrombin: except from ferrocene, they also introduced CDs and AuNPs to modify graphene.^145^ With such a composite, the sensitivity was greatly enhanced with a detection limit as low as 5.2 × 10^–18^ m. As the carrier of hereditary information, DNA has attracted the attention of researchers for many years. Bonanni et al. immobilized hairpin–DNA probes onto graphene to fabricate a sensor to detect single nucleotide polymorphism relevant to the development of Alzheimer's disease.^146^ Akhavan et al. introduced a “graphene nanowalls” structure towards single‐strain DNA electrochemical sensing.^147^ Besides, graphene was introduced into DNA sequencing. With a nanopore on the monolayer graphene, detailed electric signals can be sensed by computer when DNA runs through the pore and we can obtain the base sequence of the DNA fragment.

Just like humans, graphene‐based films also have their sensing. The gustation is categorized into five elementary tastes (sweetness, sourness, saltiness, bitterness, umami). And the graphene based‐films can be utilized for the sensing of small biomolecules, pH, metal ions, proteins and DNA. In the future, electronic tongue made by graphene‐based films may come into our lives which can be used to “taste” the flavor of foods. Besides, wireless bacteria detectors based on graphene (Figure 14 e–g) may be used in the prevention and detection of disease.^148^ We envision that the “gustation sense” would be a promising sensing for graphene‐based films due to the excellent sensitivity, wide‐range detection, and low detection limit of graphene‐based biosensors.

#### 3.1.5 Tactile—Touch Sensing

**Figure 15:**
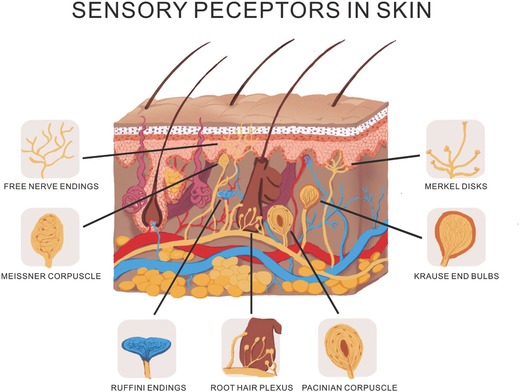
Schematic diagram of tactile sensor of skin.

The touch sense of itching caused by insect bites or allergies involves special itch–specific neurons in the skin and spinal cord. These various tactile sensors are obtained from the specialization of the nerve ending. Nerve cell can be activated when mechanosensitive ion channels are stimulated by pressure, later, electric signal spreads to the brain along the nerve fibers and then transforms into tactile sense which can be felt. Complicated tactile sensing system under bland skin is illustrated in **Figure** **15**. At present, pressure sensors are primarily based on the strength induced variation in capacitance,^149^, ^150^ piezoelectricity,^151^, ^152^ resistivity,^17^ triboelectricity.^153^ And graphene has been applied in the design of light and wearable pressure sensors. It just like the graphene films should have a sensory ability if stimulated by stress and could play a role of tactile sensor of skin in these indispensable devices. And skin‐like graphene‐based pressure sensing devices are principal for future flexible devices, such as touch screens, piezoelectric sensors, wearable electronics and triboelectric applications.

**Figure 16:**
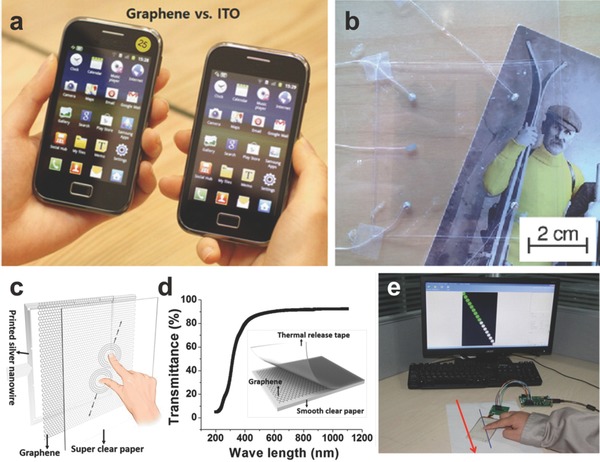
a) Photograph of a graphene‐based touch screen mobile phone (left) in comparison with an ITO‐based touch screen phone (right).Reproduced with permission.^150^ Copyright 2014, American Chemical Society. b) Sketch map of touch panels. Reproduced with permission.^154^ c) A schematic of paper based multitouch screen. d) Optical transmittance of the whole device. e) Measurement of linearity of paper touch screen. c–e) Reproduced with permission.^155^ Copyright 2016, American Chemical Society.

Iijima et al. fabricated a large‐area graphene/PET touch panel.^7^ After that, Zhi et al. reported a rod‐coating (Meyer rod) method to synthesis uniform rGO films directly on PET substrates.^9^ The thickness of the wet coating rGO film was determined by the diameter of the wire embraced around a Meyer rod. The outstanding flexibility, excellent transparency and conductivity of rGO film were also fulfilled in this work. The as‐prepared rGO/PET films were regarded as electrodes to fabricate the flexible four‐wire resistance touch screens. Then the linearity of the touch screen was detected by measuring the voltage gradient of the touch position every other 5 mm in the X and Y axis, respectively, which is comparable to that of mechanical ITO touch screens. The good linearity further confirms the stability and uniformity of the films. GO film is also functioned as the dielectric layer for sustaining heavy strains and a flexible capacitive touch pad.^149^ Moreover, the capacitor structure has been measured to exhibit high response sensitivity with fast touch rates. Cho et al. developed a rapid thermal chemical vapor deposition (RT‐CVD) for the growth of graphene films and applied to capacitive multi‐touch devices which fixed in the mobile phone (**Figure** **16**a).^150^ Such graphene films possessed over 400 × 300 mm^2^ area with a sheet resistance of 249 ± 17 Ω sq^–1^. Notably, the graphene/PET films are transparent for all visible wavelength range, nevertheless, the ITO appears to be slightly yellowish and less transparent in short visible wavelengths.

Tuukkanen et al. fabricated transparent and flexible, water‐proof piezoelectric touch panels (Figure 16b) with graphene‐based films.^154^ They obtained sufficient conductivity for this touch application using a graphene‐based ink in the solution process. Curiously, such touch panels could be applied to endure moisture and sunlight due to the sensitivity to external force. Virtually, such touch panels could be installed in appliances such as kitchen and bathroom, and even some automotive equipment. Recently, Hu et al. developed a super clear cellulose paper based multitouch screen with graphene film (Figure 16c–e).^155^ As shown in the inserted image of Figure 16c, monolayer graphene obtained by CVD method was dry‐transferred onto a piece of clear paper as a transparent electrode. Figure 16c and 16d showed the test on the linearity, optical transmittance respectively, both important parameters to reflect the performance of a touch screen. The inserted schematic shows the dry transferring process for graphene on clear paper. Due to the high electric conductivity of graphene, the device responded rapidly and accurately to the touch positions where the finger passes along a straight line (Figure 16e).

Another type of touch sensors is piezoresistive sensor that convert the pressure applied on the sensor to resistance signal.^156^, ^157^ The fabrication of piezoelectric energy harvesters with sensitivity and flexibility is essential so as to transform the mechanical energies to electricity.^158^ To further improve the electrical mobility of graphene when served as an electrode for sensitive stretchable transparent piezoelectric NGs, Kim et al. developed this device based on the decoration of graphene films by ferroelectric poly(vinylidene fluoride triuoroethylene) [P(VDF‐TrFE)] remnant polarization.^159^ The thin poly(dimethylsiloxane) (PDMS) rubber template was applied for a stretchable mechanical spring. And the flexible graphene‐based electrode is tailored for gathering electrical energy from tiny and disorder mechanical energy sources in a living environment with low magnitude and low frequency.

**Figure 17:**
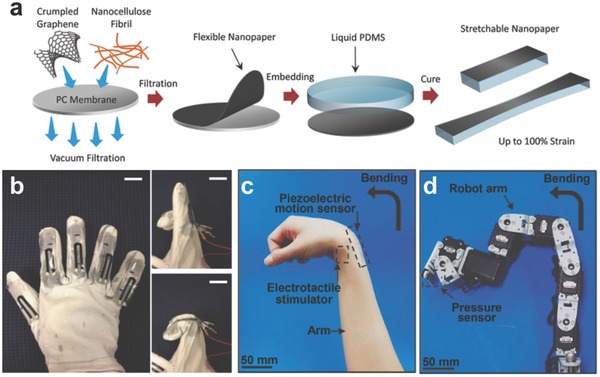
a) Schematic illustrations of the fabrication processes for stretchable graphene nanopapers. b) Photographs of the data glove with five implanted sensors. Scale bars: 2 cm. Reproduced with permission.^161^ c‐d) Representative image showing human motion to control the robot arm. Reproduced with permission.^162^

In addition, ever‐growing piezoresistive graphene‐based devices are used for detecting feeble movement or deformations of human bodies. Park et al. created a graphene‐coated polymer film as a transparent piezoresistive strain sensor.^160^ They developed a modified graphene‐based strain sensor with high optical transparency and response ability, by tuning the thickness, density of the graphene film, as well as the adhesion force between graphene and the substrate. Lee et al. presented an innovative strategy for the fabrication of stretchable piezoresistive graphene‐nanocellulose sensors with the detection limit up to 100%.^161^ The flexible nanopapers based on crumpled graphene and nanocellulose were manufactured and then installed in the elastomer matrix (**Figure** **17**a). The device includes five independent sensors embedded in a feather glove to test the deformation of fingers such as bending and stretching, as shown in Figure 17b.

Recently, wearable interactive human–machine interface (iHMI) systems have attracted attentions due to their applications in personal mobile electronics and the internet. Kim et al. developed a transparent and stretchable iHMI system made of wearable mechanical sensors and stimulators.^162^ The process, capturing target objects via the robot, can be monitored by a piezoelectric pressure sensor implanted on the gripper. The successful performance of this mission activates the electrotactile stimulator set on the skin of the user. Conformal integration of flexible piezoelectric sensors on human skin decreases artifact signals and increases the of precision machine control. Figure 17c–d showed representative images of the synchronized motion of a robot controlled by the human wearer (i.e., bending a robot arm by the controlling motion of a human). Such high transparency, excellent performance, and low power consumption as well as mechanical deformability profit from the conductive/piezoelectric graphene heterostructures.

**Figure 18:**
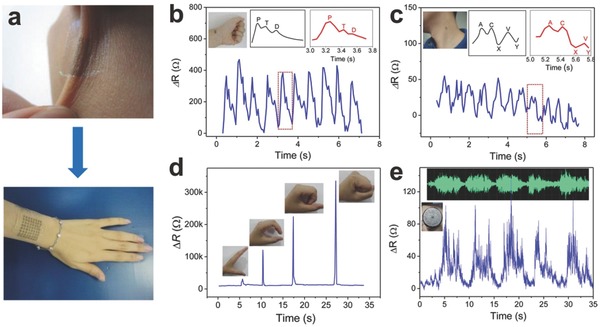
a) Conformal adhesion of a GWF‐based strain sensor on the human skin. b) Measured relative resistances of pulse and c) jugular venous pressure measured in real time. d) Resistance response at the successive stages of making a fist. e) Recognition of a sound signal using the GWF strain sensor. Reproduced with permission.^17^ Copyright 2015, American Chemical Society.

Zhu et al. reported a highly strain sensitive graphene‐based electromechanical sensor with a gauge factor of 500 below 2% and 104 over 8% through tailoring the graphene integral network configuration, crack formation, and propagation mode.^17^ GWFs‐based strain sensors with the woven fabric structure are more sensitive than a flat network structure of patterned graphene. As **Figure** **18**a demonstrates, such sensor was connected to a deformed skin surface with a visible wrinkle below. Figure 18b shows the possibility of employing the GWF as a pulse detector by ordering the sensor around the wrist. A typical biphasic waveform of the jugular venous pressure could be detected with a reproductive waveform shape, which carried valuable information about the human physical condition, as illustrated in Figure 18c. Figure 18d shows the resistance change of a GWF attached to the finger joint during the motion of making a fist. And sound vibration indicated another kind of mechanical wave of pressure, of which the perception and processing was an overall part of human intelligence. The inset in Figure 18e shows the audio measuring process with music carried out by a loudspeaker and the GWF‐based strain sensor linked to the surface of the loudspeaker's vibrating membrane.

In short, skin‐like graphene‐based pressure sensing devices have aroused tremendous attention for flexible devices, especially touch screens, piezoelectric sensors and wearable devices. It just like the graphene films that could readily distinguish various strain levels of outer signals. Furthermore, these applications toward going light, wearable, self‐powered electronics will be required in the future.

### 3.2 Reflexes

**Figure 19:**
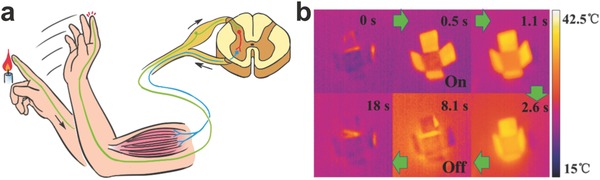
a) The transfer path way of human reflex. b) The reflex of graphene device with and without light illumination. Reproduced with permission.^166^ Copyright 2015, American Association for the Advancement of Science.

Reflex action is a spontaneous and momentary movement in response to a stimulus (**Figure** **19**). In other words, the organism will move or act when get stimulated. As analog to graphene actuated devices, they will do the corresponding action when stimulated. The stimulating source can be categorized according to photothermal actuators,^163^, ^164^, ^165^, ^166^ water actuated devices,^167^, ^168^ and electromechanical actuator.^169^, ^170^, ^171^

When an organism is exposure to the light or thermal radiation, it will tend to get closer or further, which is similar to the photo‐thermal actuated graphene devices. The transparent soft actuators were successfully fabricated by using large‐area graphene, which showed ultrasensitive responses.^164^ And new prototype soft robot models were successfully designed when infrared light was served as driving energy. Graphene–elastin composite hydrogel actuators controlled by light was also studied by Lee et al. By interfacing genetically engineered elastin‐like polypeptides with rGO sheets, near‐infrared light (NIR)‐driven hydrogels actuators (HAs) was synthesized.^165^ When changing the location of a night‐time ozone profile laser spot, the fingers of a hand‐shaped hydrogel can achieve bending or unbending and a light‐driven crawler was successfully realized.

**Figure 20:**
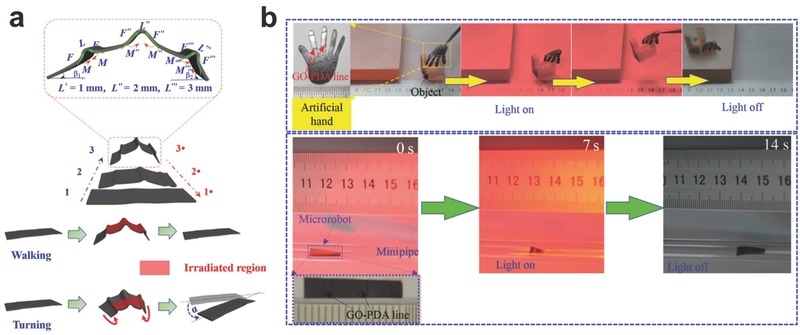
The hand and wormlike auto devices. a) The mechanism of the wormlike walking device. b) Optical images revealing the hand holding and the “microrobot” driven by light irradiation.Reproduced with permission.^166^ Copyright 2015, American Association for the Advancement of Science.

By elevating the temperature of the materials, different degree expansion was carried out to make the devices move. The rGO‐CNT/PDMS bimorph photoactuators were introduced by Chen et al.^163^ After curing at high temperature, thermal stress was induced into the rGO‐CNT/PDMS bimorph. And the pseudo‐shape‐memory feature of the rGO‐CNT/PDMS also makes it possible to be photoactuators. As a result, a series of light driven devices, including smart “roller blinds”, smart “box” and crawler‐type “robot” were constructed. Even more interesting is that origami‐inspired active graphene‐based paper has also been constructed, which also achieved programmed gradients both in vertical and lateral directions.^166^ In this work, the function‐designed GO was used as building blocks to construct graphene self‐folding paper. The graphene paper can achieve walk and turn a corner when remote‐controlled by light or heating. By designing the programmed gradients, a cross‐shaped graphene paper can fold into a box under NIR light, and the response was very fast with a short time of 200 ms, as shown in **Figure** **20**. Moreover, the wormlike walking device was carried out by designing the GO‐polydopamine (PDA) width (Figure 20a). In addition, it also can grasp and hold objects five times heavier than itself and serve as a microrobot which can be remotely operated inside a closed and minispace (Figure 20 **b**). This smart widges fabricated by designing graphene was operated under IR laser control which was not required any additional external stress.

Similar to humans, the graphene actuators responded not only to the photic and thermal stimuli, but also to the watery and electric stimuli. A single‐fiber walking robot was successfully achieved by utilizing graphene/graphene oxide (G/GO) fibers, which prepared by partial asymmetric positioning laser reduction of freshly spun GO fibers.^168^ In addition, a new type of moisture driven rotational motor was also carried out.^167^ It was fabricated by reconstructing the configuration of graphene through the rotary processing of freshly spun GO fiber hydrogel, forming helical arrangement of graphene sheets along the GO fiber. This GO rotational motor can achieve a rotary speed as high as 5190 rpm under humidity alternation. And the tensile expansion was of 4.7%. It also can serve as humidity switches and humidity triggered electric generators.

By asymmetrically modifying the surface of monolithic graphene film, a novel graphene electrochemical actuator was successfully achieved by Dai et al.^171^ The front and the back of the graphene film were treated by hexane and O_2_ plasma respectively, and the actuation motion was induced through asymmetric electrochemical charging and discharging. Graphene–PDA bimorph actuator is also designed by using a simple yet versa tile method.^169^ The conformation of PDA would change when external stimuli was induced. After combining the respective particular features of graphene and PDA, the actuator shows a commendable actuation performance exceeding to the conventional electromechanical actuators.

## 4 Conclusion

Sensing is the response to the objective characteristics (sound, color, smell, etc.), or the stimulation from the material world directly affecting the sense organs of the organisms, such as light cause the vision and sound waves give rise to the hearing. As we known, there are no authentic sensations in the inorganic world except for the similar features with sense, which is the pure chemical or physical reflection. Since life appeared on earth, simulated sensitivity including the sprout of feelings comes into being. Humans have a multitude of senses, wherein sight, hearing, taste, smell, and touch are the five traditionally recognized senses. With the stimulation from external world, the corresponding signals would be transduced into electroneurographic signals. In analogy with this theory, we were surprised to see that graphene‐based films exhibit sensing and reflex in similar features to human. Due to its superior characters such as high conductivity, HCPUA, high specific surface area and flexibility, graphene was increasingly utilized to sense the light, sound, pressure and so on. Based on our brief journey to graphene and graphene ramifications, it can be concluded that graphene is a unique two‐dimensional material, offering an extensive range of opportunity for human‐like stimulated sensitivity. That is the very reason makes us lift our eyes to the broad horizon ahead, wishing one day to realize the high‐fidelity artificial organs. However, challenges still remain regarding the ultimate features achievable via operable techniques.

Like nerve conduction velocity effected by neurotransmitter, it is not a stretch to think of at least a relation between the human‐like stimulated sensitivity and the quality of the graphene films. CVD growth and solution processing for graphene films attract more attention for their scalability, but domain or sheet size and defect controls are the pivotal issue. The sheet resistance of graphene films is related to the synthesis method. The ideal *R*_s_ ≈ 30 Ω sq^–1^ of graphene in theory is attractive but hard to achieve. As for the current research situation, the graphene‐based film is still about 1 order of magnitude higher in sheet resistance than ITO. This means that graphene has to be either doped or hybridized with other material, decreasing its sheet resistance. It's worth noting that the instability of graphene–dopant interactions in air and under thermal loading limits its lifetime and thus causes an increase of *R*_s_. The graphene hybridized with other materials, such as CNTs and metal nanowires, can facilitate the low‐cost and high‐speed fabrication of graphene‐based films.

Before applied to the artificial organs, graphene films should be first utilized for operable electronical devices. Similar with the light‐sensitive human eyes, graphene film serving as the electrode for SC or PD converts light signals into electronical signals. The photoelectric conversion efficiency is affected by the conductivity and transmittance of the graphene film. According to the thin and flexible graphene film, it is reasonable to be applied to the sound generators, such as a microphone, an earphone and a recorder. What is crucial in sound generators is the ultra‐small HCPUA than other materials. And it worth noting that the defects or functional groups on graphene films facilitate the adsorption of various molecules, both small gas molecules and biomolecules. Working like the nose and tongue of humans, the graphene films in gas sensors or biosensors adsorb molecules and react with them, as a result of the changes in conductivity or other properties. Like the touch of humans, graphene films can also sense pressure, friction, and touch. Serving as the component of the touch screen, wearable devices and piezoelectric devices, graphene films exhibit high sensitivity and flexibility. Apart from these five traditional senses mentioned above, graphene films also demonstrate mechanical motion once stimulated by thermal radiation, light, or humidity, to name a few. Although dramatic progress has been made in this field, great challenges still exist in commercializing applications of graphene. The challenges can be summarized as follows: (1) make a balance between low sheet resistance and extremely high transmittance. (2) the preparation of large‐area devices for more emerging applications such as artificial skins and intelligent interfaces.

Except for the general senses, there are several non‐traditional senses of human, such as balance, acceleration, temperature, kinesthetic sense, and pain. On account of this, pushing into new territories of human‐like senses gives a new estimate. In addition, to realize high‐fidelity artificial organs, we need to develop the integration of multiple human‐like senses of graphene films. And, once achieved, we firmly believe that graphene will raise a revolutionary of human‐like senses and reflexes, paving its flourishing way to future commercial devices.
